# Primary Midgut Volvulus: An Unusual Case of a Young Adult Necessitating Extensive Bowel Resection

**DOI:** 10.7759/cureus.54472

**Published:** 2024-02-19

**Authors:** Ibrahim El Nogoomi, Rania Jumah, Khadijah O Zaidan, Ammar Agha

**Affiliations:** 1 General Surgery, Al Kuwait Hospital, Sharjah, ARE; 2 General Practice, Al Kuwait Hospital, Sharjah, ARE; 3 Surgery, Al Kuwait Hospital, Sharjah, ARE

**Keywords:** case report, acute abdomen, intestinal obstruction, small bowel ischemia, primary small bowel volvulus

## Abstract

Primary small bowel volvulus (SBV), commonly known as midgut volvulus, is an uncommon condition in which the small intestine rotates around its own mesenteric axis. This case report details the diagnostic and management challenges encountered in a rare presentation of primary SBV in a previously healthy 19-year-old male. Our patient presented with acute abdominal pain, vomiting, and signs of shock, prompting urgent medical attention. He was sent for exploratory laparotomy and underwent extensive resection of the gangrenous bowel. Diagnosis involved an abdominal computed tomography scan revealing the characteristic “whirl sign.” According to the World Society of Emergency Medicine, surgical intervention should be done to address the SBV through resection of the gangrenous bowel segments. Despite efforts, the patient’s prognosis remained guarded, necessitating ongoing supportive measures. This case highlights the complex challenges associated with primary SBV, emphasizing the need for continued research to enhance diagnostic precision and refine management strategies.

## Introduction

Volvulus is defined as the twisting of a loop of the bowel on its mesentery and is one of the most common causes of intestinal obstruction. It presents as an acute abdomen and the symptoms may be due to narrowing of the bowel itself, strangulation of the blood supply, or both. This can lead to ischemic necrosis of the bowel, indicating the necessity of prompt diagnosis and surgical intervention [1].

Primary small bowel volvulus (SBV) is an extremely rare condition in adults with no underlying anatomic abnormalities or known predisposing factors. Secondary SBV is more common with postoperative adhesions representing the main cause [2].

SBV is considered a rare cause of small bowel obstruction in Western countries, comprising 1-6% of cases. However, it accounts for 20-35% of small bowel obstructions in Asia, Africa, and the Middle East [3].

In this case, we present a healthy young adult with primary SBV. The patient had only 30 cm of viable jejunum remaining after all the remaining distal gangrenous small bowel was removed until reaching the ileocecal valve.

## Case presentation

A 19-year-old man presented to the emergency department at 7:00 am with a history of abdominal pain and multiple episodes of vomiting since 10:00 pm. The pain started suddenly after having a meal. The pain was generalized throughout the abdomen with the maximum point at the epigastric. Moreover, it was colicky and becoming worse. No aggravating or alleviating factors were reported. The pain was associated with multiple episodes of projectile vomiting of clear fluid. He had not eaten or drank since 10:00 pm. As his condition worsened in the morning and he became drowsy and very sick, he came to the emergency.

There was no reported history of trauma, urinary symptoms, change in urine color, melena, recent weight loss, change in bowel habits, fever, or rash. There was no significant family history of chronic medical conditions. There was no previous hospital admission or any past medical history of asthma, hypertension, and diabetes. He had not undergone any surgical intervention throughout his lifetime. He did not have any allergies and was not on any medication. He was living in a flat with his roommate. He had recently traveled to India 14 days before his presentation, and there was no history of smoking, alcohol, or illegal substance use.

Upon arriving at the emergency his vitals were a temperature of 36.6°C (axillary), heart rate of 118 beats/minute (peripheral), respiratory rate of 18 breaths/minute, blood pressure of 85/52 mmHg, and SpO_2_ of 96% (supine). On examination, the patient appeared very ill. He was conscious but disoriented and agitated. He seemed severely dehydrated with sunken eyes. The patient was sweating and had clammy skin. He was not jaundiced or cyanosed. Upon chest examination, the patient had equal air entry bilaterally, normal S1 and S2, with tachycardia. Abdominal examination showed gross distention with generalized tenderness, no guarding, and no rigidity, in addition to the normal hernial orifice and no organomegaly. Per rectal examination was normal. The genitalia were also normal. He was able to move all limbs. Rapid arterial blood gas (ABG) analysis was done, which showed a pH of 6.94, pCO_2_ of 22.0 mmHg, pO_2_ of 363 mmHg, and HCO_3_ of 4.6 mEq/L, showing metabolic acidosis. Lab results showed a white blood cell count of 24.63 x 10^3^/µL, random blood sugar of 16 mmol/L, and creatinine of 323 µmol/L. The rest of the results are presented in Table 1.

**Table 1:** Laboratory studies on admission. PT: prothrombin time; APTT: activated partial thromboplastin time; INR: international normalized ratio; CK: creatine kinase; CRP: C-reactive protein; ALT: alanine aminotransferase; AST: aspartate aminotransferase; AP: alkaline phosphatase

Complete blood count		Basal metabolic panel and urine analysis		Coagulation and others		Liver and renal function test		Arterial blood gases	
Hemoglobin (g/dL)	14.3	Sodium (mmol/L)	148	PT (seconds)	18.5	Total bilirubin (µmol/L)	16.4	pH	6.94
Hematocrit (%)	24.3	Potassium (mmol/L)	5.26	APTT (seconds)	47.3	Total protein (g/L)	67	pCO_2_ (mmHg)	22
White blood cells (×10^3^/µL)	24.63	Chloride (mmol/L)	109	INR	1.63	ALT (IU/L)	90	pO_2_ (mmHg)	363
Neutrophils (%)	68.80	Random glucose (mmol/L)	16	Troponin-I (ng/L)	597.16	AST (IU/L)	96	Bicarbonate (mEq/L)	4.6
Lymphocytes (%)	28.80	Urine ketones	Negative	Albumin (g/L)	35.4	AP (IU/L)	117.5	Oxygen saturation (%)	96
Platelets (×10^3^/µL)	408			Total CK (IU/L)	477	Uric acid (mmol/L)	9		
				Amylase (IU/L)	47	Creatinine (µmol/L)	323		
				CRP (mg/L)	3.3	Urea level (mmol/L)	7.68		

In the emergency department, the patient received 3 L of normal saline and 100 mL of sodium bicarbonate, along with pantoprazole 40 mg intravenously and piperacillin-tazobactam. His ABG results after sodium bicarbonate infusion were a pH of 7.168, pCO_2_ of 31 mmHg, pO_2_ of 98 mmHg, and HCO_3_ of 12 mEq/L. A nasogastric tube was inserted. There was minimal bleeding. Surgical and medical oncalls were called.

**Figure 1:**
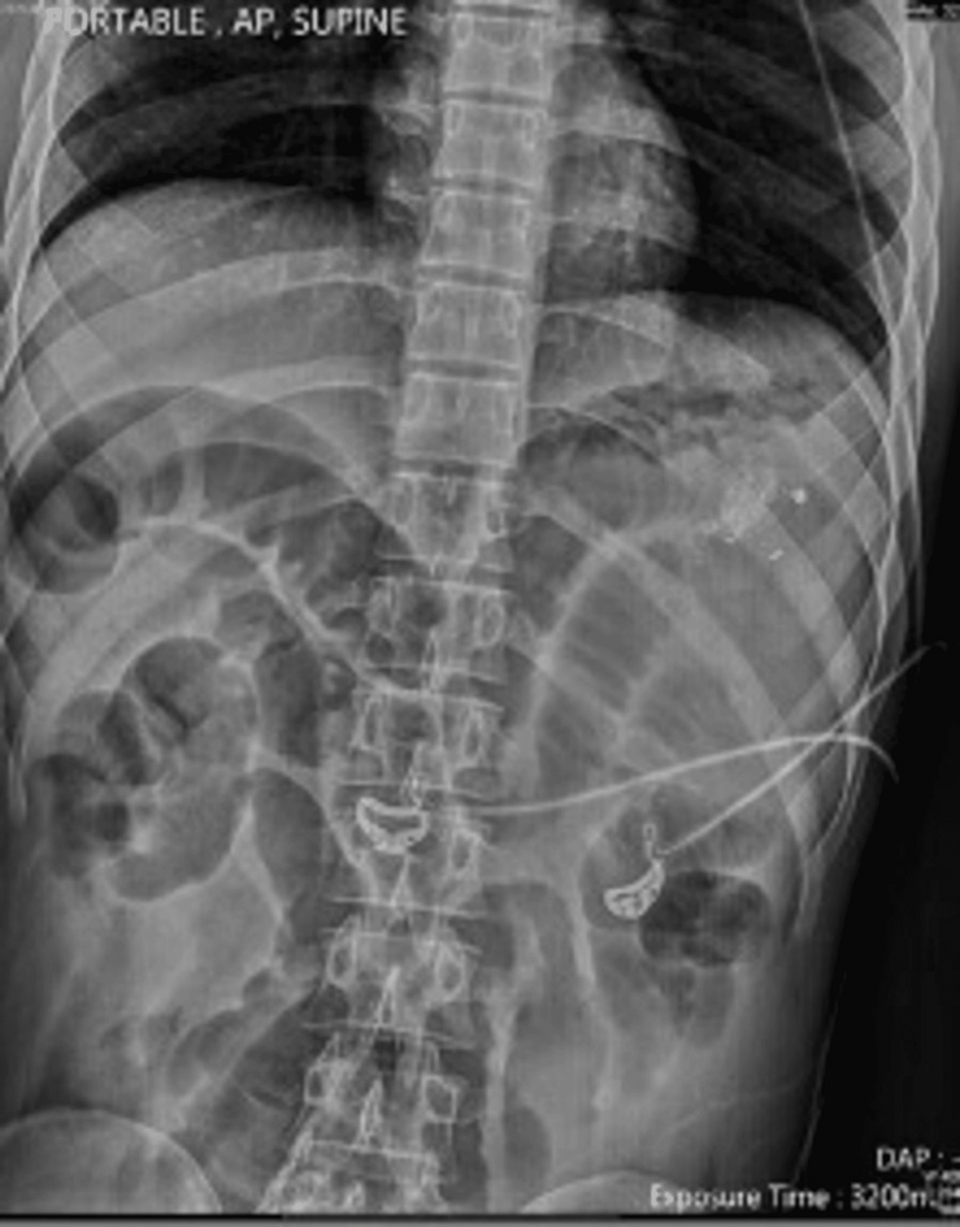
Supine X-ray. Dilated bowl loops can be noted. The patient was not able to undergo an X-ray in an erect position because he was unstable.

On supine abdominal X-ray, there were no calcific foci, aberrant air-fluid levels, or free intra-abdominal air visible. Non-specific moderate gas distension was observed in the bowel gas pattern (Figure 1).

**Figure 2:**
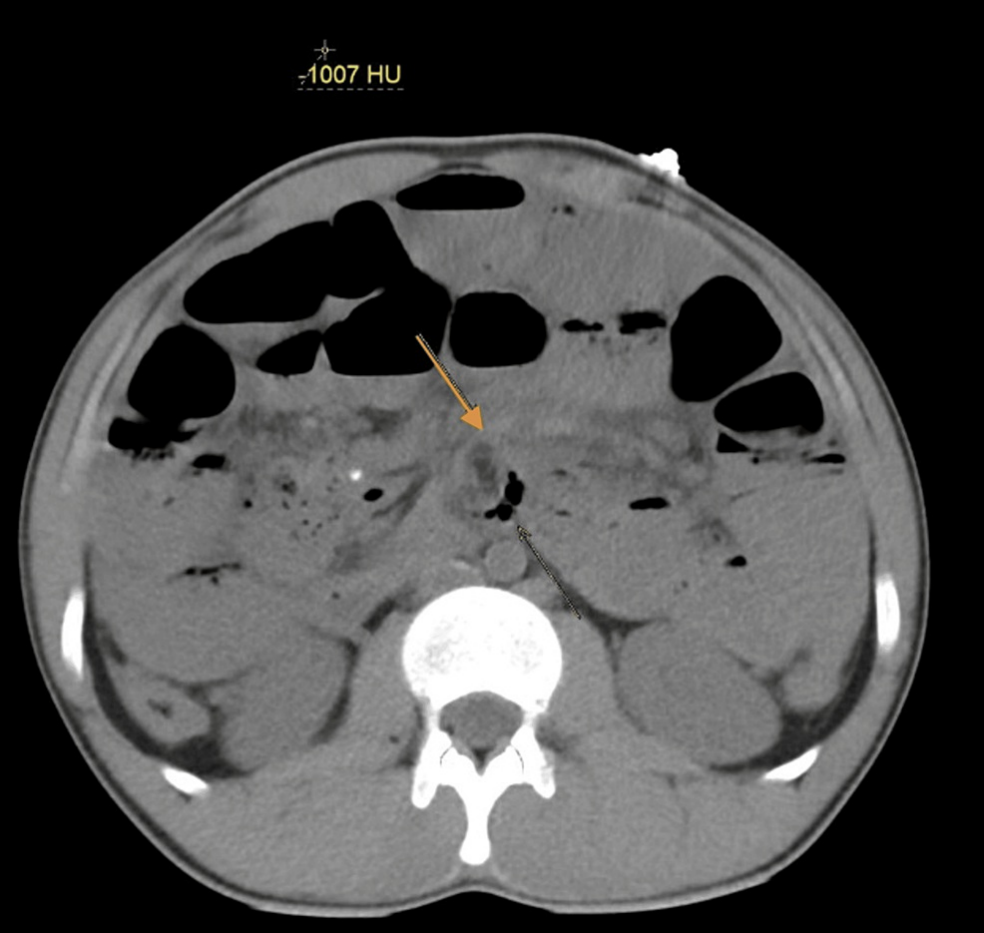
Axial plane. The arrow is pointing to the whirl sign.

**Figure 3:**
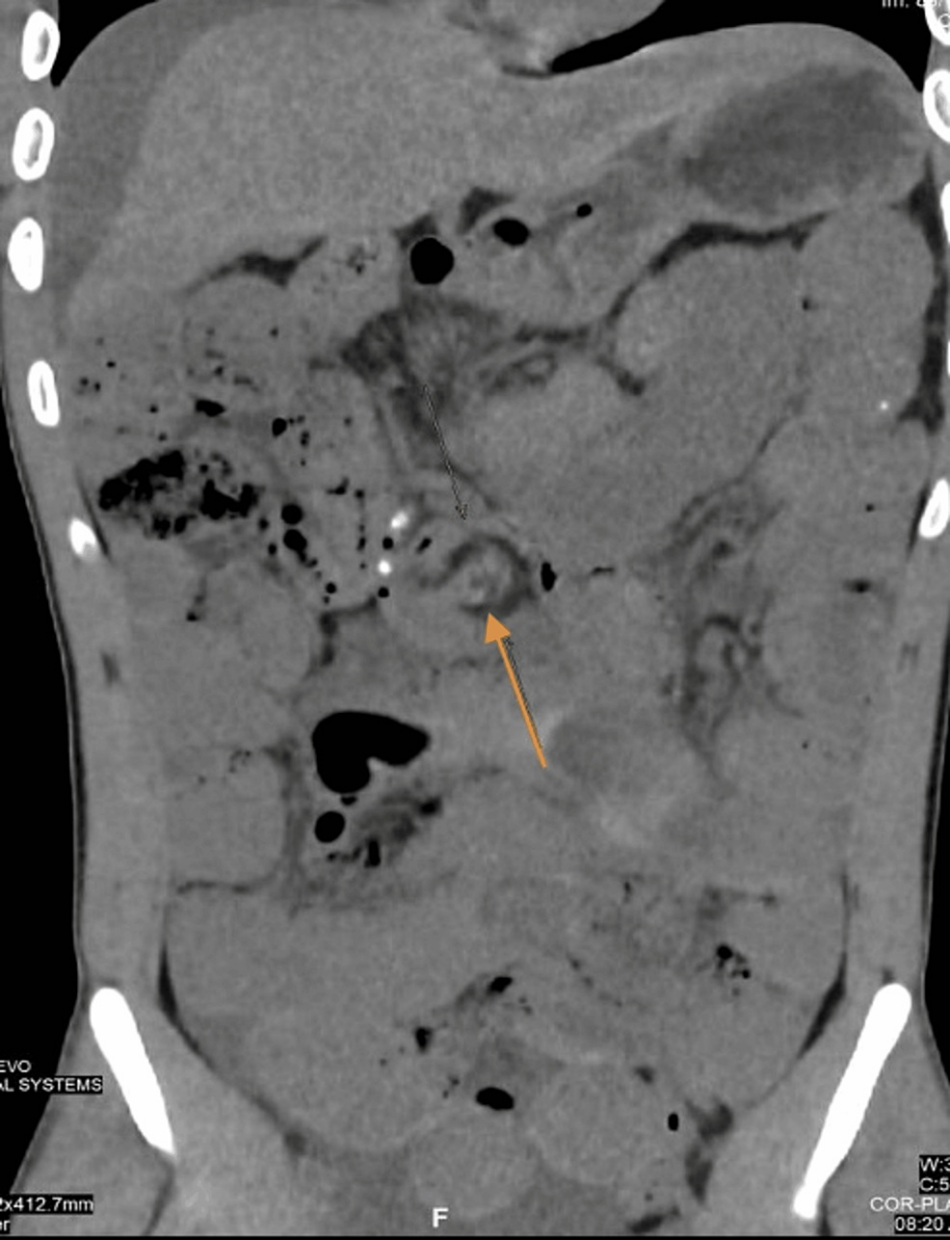
Coronal plane. Coronal plane showing the whirl sign with an edematous lymph node around it.

**Figure 4:**
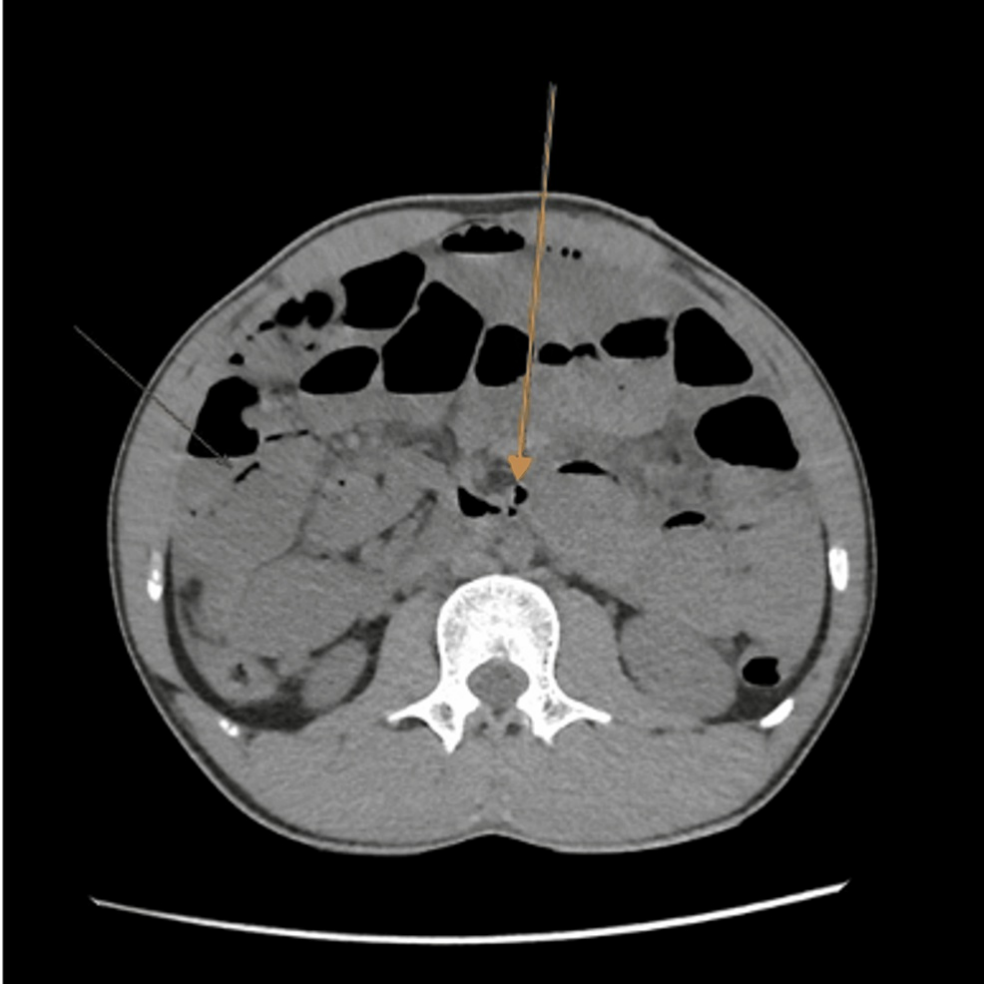
Sagittal plane. Sagittal plane showing intramural bowel gas, also known as pneumatosis intestinalis.

**Figure 5:**
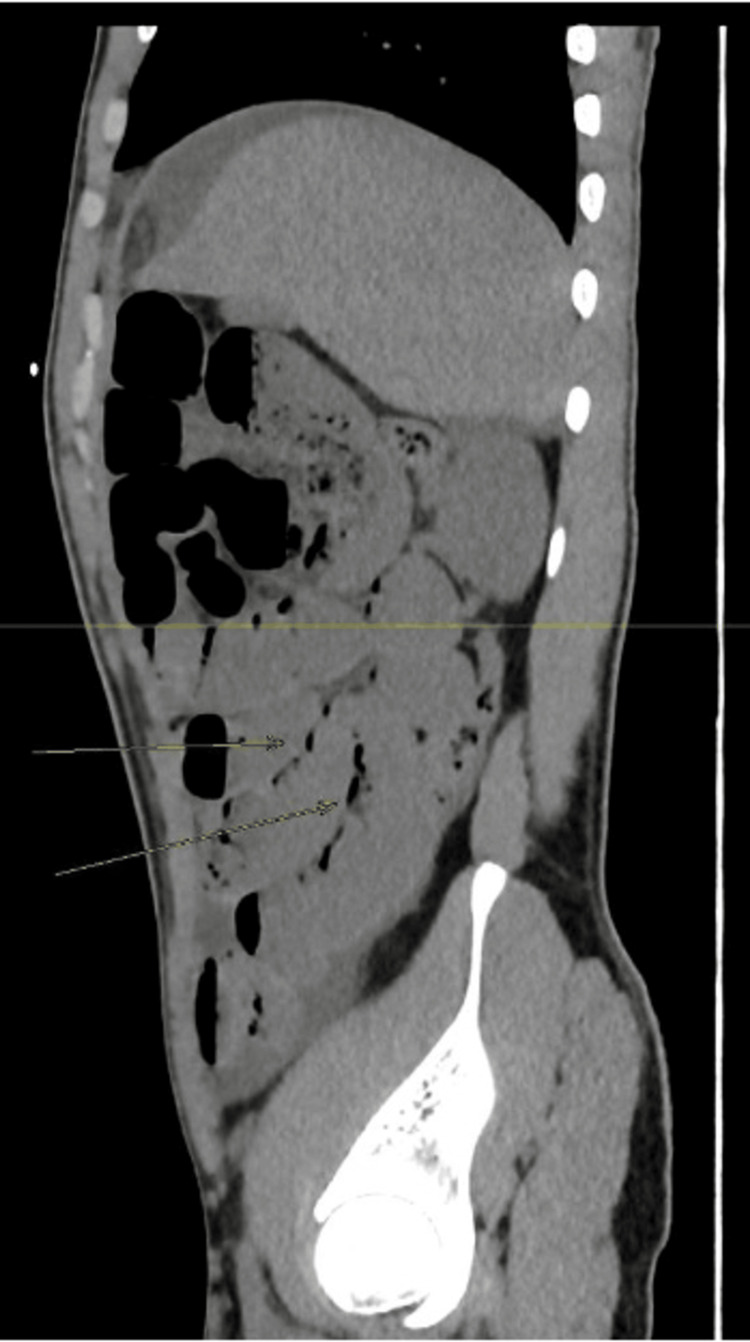
Axial plane. Arrows are pointing to intramural bowel gas, also known as pneumatosis intestinalis.

After volume resuscitation, the patient underwent an abdominal non-contrast computed tomography (CT) scan of the abdomen to exclude surgical emergencies, which showed dilated loops of small bowel filled with fluid that extended to the terminal ileum. The largest diameter of the dilated loops was 3.6 cm. A distinct transition zone was observed at the ileocecal junction between the dilated and non-dilated loops, and a positive small bowel feces sign was also seen. There was no dilatation in the rectum, colon, or appendix lumen, and no evidence of pneumoperitoneum, although a little pelvic-abdominal fluid collection with a comparatively high-density attenuation value was noted. The whirl sign was detected with edematous lymph nodes surrounding it (Figures 2, 3). Furthermore, pneumatosis intestinalis was noted, which is intramural bowel gas and is an indication of bowel ischemia (Figures 4, 5).

In addition, the intraperitonial space surrounding the liver showed mild ascites. The gallbladder, spleen, pancreas, adrenal glands, and both kidneys appeared normal in this non-contrast study. Subsequently, the patient was recommended to undergo a contrast-enhanced CT scan of the abdomen to assess the possibility of bowel ischemia; however, the radiologist refused this request due to the patient’s critical renal parameters, which posed a risk of permanent renal dysfunction requiring lifelong dialysis. The radiologist recommended stabilizing the patient’s condition and consulting with nephrologists regarding his renal status before considering a contrast-enhanced study.

During esophagogastroduodenoscopy, examination of the stomach, D1, and D2 regions showed no altered mucosal tissue, but there was evidence of fresh bleeding. Following attempts to alleviate the patient’s condition through flushing with water and suctioning, no specific lesion, vascular abnormality, or ulceration was identified in these areas. However, upon progressing beyond the D2-D3 segment, mucosal edema, congestion, and ulceration were observed, indicating possible bowel ischemia. A diagnostic abdominal aspiration guided by ultrasound imaging of the abdomen was performed, which yielded 20 mL of aspirated blood, highlighting the presence of a hemorrhagic component in this clinical case.

**Figure 6:**
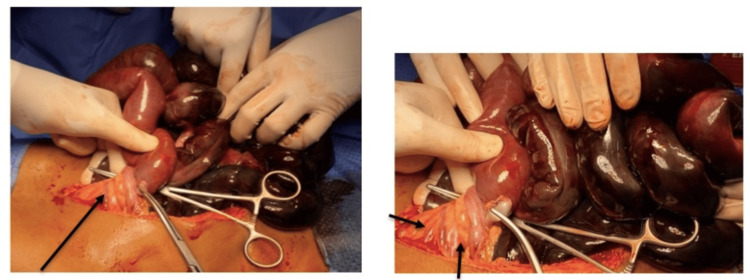
Necrotic bowel. Twisting of the small intestine around the root of the mesentery can be noted.

**Figure 7:**
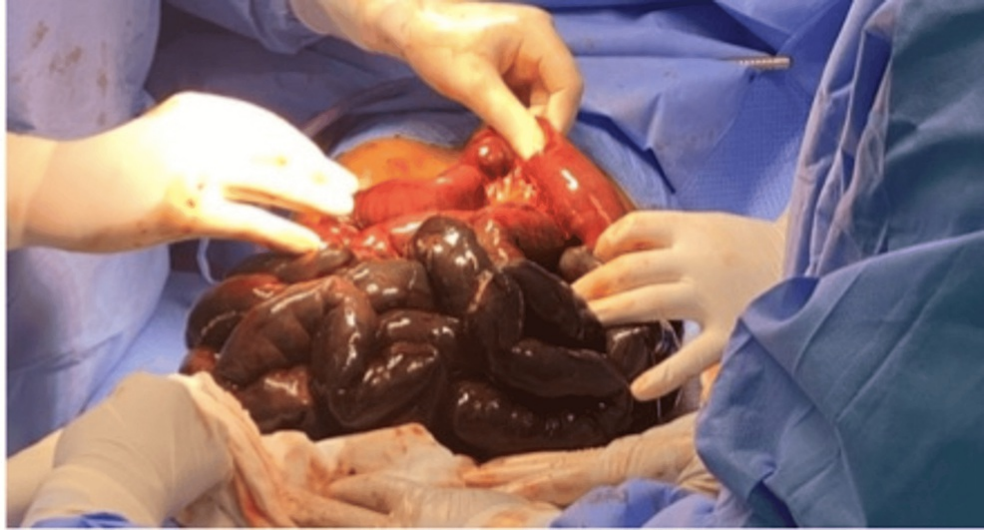
Gangrenous small bowel segment.

The patient was sent for exploratory laparotomy due to SBV. The surgical technique involved a midline laparotomy incision. Findings showed twisted small bowel loops around the root of the mesentery, indicative of volvulus, as well as a significant segment of gangrenous small bowel measuring 175 cm distal to the duodenojejunal junction and 10 cm proximal to the ileocecal junction, with the total gangrenous extent measuring 390 cm. Additionally, a substantial volume of free intraperitoneal fluid was observed (Figures 6, 7). Surgical excision of all gangrenous bowel segments was done, with secure suturing of the mesentery cut margins using 2/0 Vicryl locking sutures, as well as thorough clearance of the bowel content, with saline filling the stomach and flushing of any blood present in the small bowel. The healthy ends of the small bowel were anastomosed and closed using a linear stapler. Surgical irrigation of the abdominal cavity was performed with saline, and an 18 Fr Nelaton drain was placed in the pelvis. The abdominal sheath was closed with a continuous Prolene loop suture.

**Figure 8:**
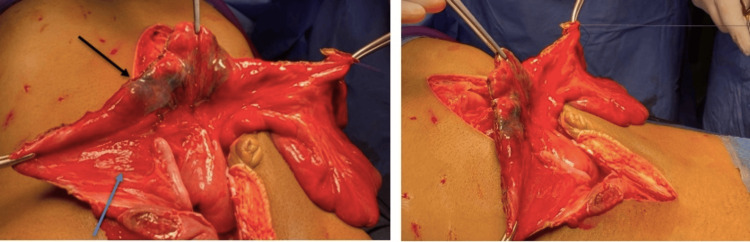
Images after the first resection. The images were taken after the first resection showing the appendix (blue arrow) and a thrombosed mesenteric artery (black arrow), which led to the patient undergoing a second laparotomy with resection of the gangrenous bowel that was remaining (30 cm).

**Figure 9:**
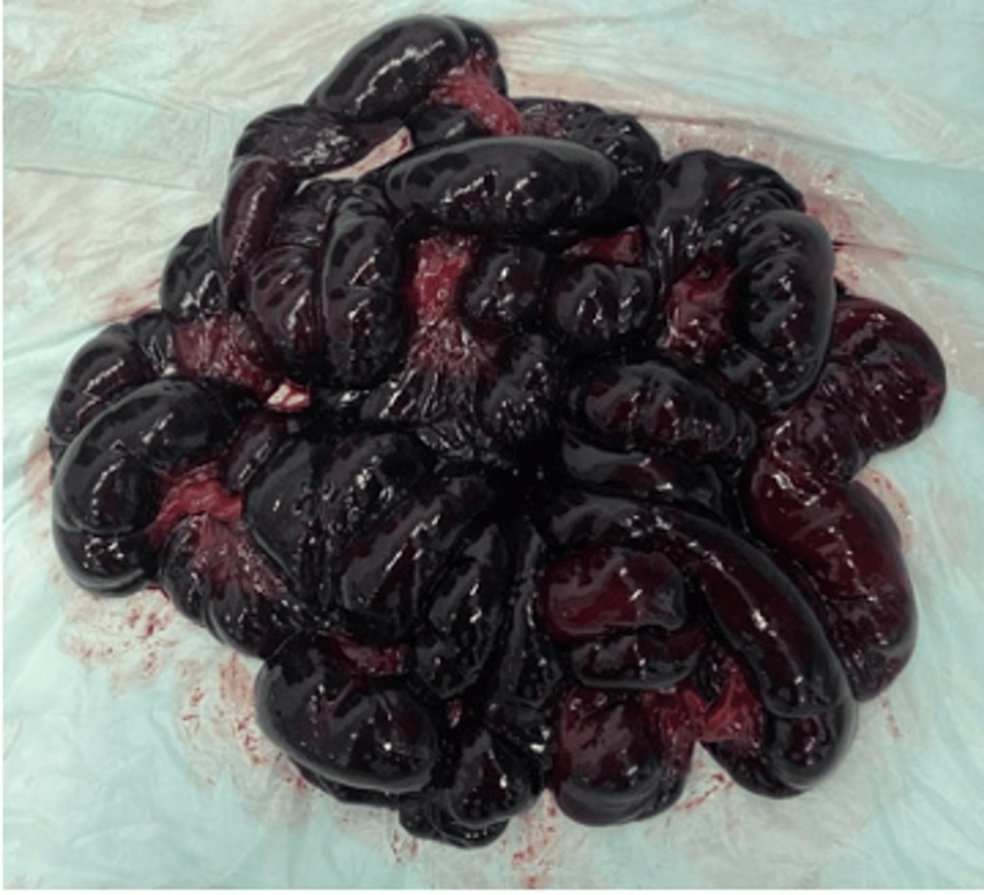
The resected gangrenous bowel.

The skin was left open for a relook laparotomy which was performed 48 hours later (Figures 8, 9). The patient was shifted to the intensive care unit for close-up observation and further management from the medical and nephrology team. He was unconscious, with guarding of the abdomen, and febrile with anuria. The midline laparotomy wound was reopened and all gangrenous bowel segments and appendix were carefully resected, leaving a 30 cm segment of healthy small bowel from the duodenojejunal junction. The cecum was mobilized and freed from lateral wall adhesions. A side-to-side anastomosis was performed between the cecum and the jejunum using 3/0 Vicryl sutures. The stomach was filled with saline through a nasogastric tube and advanced to the small bowel until it passed the anastomosis, with no evidence of leakage from the anastomotic site. The abdominal cavity was thoroughly irrigated with saline, and a corrugated drain was placed within the pelvic cavity. The abdominal sheath was closed with a continuous Prolene loop suture. The skin was managed using a skin stapler. No further surgical interventions were deemed necessary for the patient at this stage.

He was shifted to the surgical intensive care unit where his prognosis remained guarded with a Sequential Organ Failure Assessment score of 10, Acute Physiology and Chronic Health Evaluation II score of 33, and more than 73% rate of mortality, necessitating ongoing supportive measures including ventilation, hydration, thermal regulation, and inotropes for recovery and survival. As he was also anuric, he was on daily dialysis. Five days postoperatively he started having acute respiratory distress syndrome. Due to complications and multiple organ failure, a do-not-resuscitate discussion was held.

After 29 days of admission, the patient passed away due to multiple complications. The leading cause of his death was ARDS. The patient had a cardiac arrest and was not resuscitated.

## Discussion

Volvulus is defined as the twisting of a bowel loop on its mesentery and is one of the most common causes of intestinal obstruction. Primary volvulus manifests without obvious anatomical abnormalities or predisposing factors and the precise etiology remains unknown. Primary volvulus can manifest in various gastrointestinal locations, including the small intestine, as in our case, stomach, or large intestine [1,2].

Short bowel syndrome is a certain outcome after extensive resection of the small bowel, with patients who are left with only 180-200 cm of viable bowel often developing it. In our patient, only 30 cm of viable bowel remained after the duodenojejunal flexure. Hence, he was expected to develop an irreversible chronic intestinal failure as he was under the category of less than 60 cm remaining viable small bowel with jejunocolonic anastomosis requiring the need for lifelong parenteral nutrition. While parenteral nutrition can support for some time, it is not a long-term solution due to potential complications such as liver failure, hyperglycemia, and infections.

The small bowel is important in terms of absorption as there are some minerals and vitamins that are only absorbed in some areas of the intestines. The first 100 cm of the jejunum is responsible for absorbing most nutrients, while the last 100 cm of the ileum is responsible for absorbing bile salts and vitamin B12. Unfortunately, our patient had only 30 cm of the jejunum remaining and nothing left of the ileum [4,5].

Diagnosing SBV poses a challenge owing to its non-specific presentation [6]. Patients lacking a history of previous surgeries typically exhibit symptoms such as acute abdominal pain, agitation, abdominal tenderness, and, in severe cases, shock [7]. Abdominal CT is a diagnostic tool of choice in facilitating diagnosis by looking for the whirl sign. This sign indicates a visible swirl of soft tissue and fat in the middle of the abdomen, with nearby loops of bowel wrapped around twisted intestinal vessels [3,8,9].

The management approach for primary SBV once suspected strictly adheres to the guidelines established by the World Society of Emergency Surgery [10]. Timely surgical intervention is crucial to prevent new development of intestinal necrosis, ischemia, and perforation, thereby reducing both morbidity and mortality. Preoperative supportive measures involve the use of a nasogastric tube, indwelling catheter, maintenance of strict fluid balance, venous thromboembolism prophylaxis, and appropriate intensive care if required. While laparotomy is considered the gold standard treatment, laparoscopy is emerging as a viable therapeutic alternative [11].

The standard procedure involves devolvulation with concurrent resection of non-viable bowel. Controversy surrounds the various interventions aimed at preventing the recurrence of primary SBV. Detorsion alone carries a recurrence risk of 30% [12]. Although enteropexy is a therapeutic option, it is associated with the potential risk of fistula formation [13]. Mesenteric plication, as opposed to bowel plication, increases the risk of vascular injury [9,10]. Some studies have proposed systematic prophylactic surgical resection, although this is linked to elevated postoperative morbidity and extended hospital stays [14]. Unfortunately, there is insufficient evidence supporting the superiority of any of these strategies. Further research and clinical exploration are warranted to establish optimal approaches for managing primary SBV and preventing its recurrence.

One of the complications in our patient was acute kidney injury (AKI). AKI is a sudden and often reversible decline in kidney function. Common causes include prerenal factors impacting blood flow to the kidneys. Intrinsic renal causes involve direct damage to kidney tissues, exemplified by conditions such as acute tubular necrosis or glomerulonephritis. Postrenal factors include urinary tract obstructions. Additionally, systemic issues such as sepsis, infections, and certain medications contribute to AKI, emphasizing the diverse etiology of this critical medical condition. Therefore, the theory behind this patient’s acute renal injury is related to the gut-kidney axis. The gut-kidney axis refers to the bidirectional communication and interaction between the gastrointestinal system and the kidneys, as it involves various mechanisms, including the influence of gut microbiota, inflammatory responses, and the impact of gut-derived factors on renal function. First, the acute hemodynamic changes due to excessive vomiting and gut ischemia led to systemic hypoperfusion and left the patient in a hypovolemic shock and electrolyte imbalance. Second, gut ischemia and necrosis triggered an inflammatory response which may have further contributed to the kidney injury. Furthermore, the risk of bacterial translocation from the compromised gut can introduce endotoxins into the bloodstream, contributing to inflammation and further impacting the kidneys. In conclusion, AKI and gut ischemia share a complex interplay, where one can influence the other through systemic processes. Recognizing and managing gut ischemia is crucial in preventing or mitigating AKI [15-17].

## Conclusions

SBV is a very rare condition in adults, referring to the abnormal twisting of the small bowel around its mesenteric axis leading to obstruction. In general, clinical symptoms and diagnosis lack specificity. Intestinal necrosis and complications can be prevented by early detection which can be evoked with a CT scan and prompt surgical intervention to restore intestinal blood flow, resection, and anastomosis as necessary. Several other methods to approach the management and recurrence of SBV have been discussed. Thus far, all those options have been non-consensual.
